# The issues with tissues: the wide range of cell fate separation enables the evolution of multicellularity and cancer

**DOI:** 10.1007/s12032-020-01387-5

**Published:** 2020-06-13

**Authors:** Emma U. Hammarlund, Sarah R. Amend, Kenneth J. Pienta

**Affiliations:** 1 Translational Cancer Research, Department of Laboratory Medicine, Lund University, Lund, Sweden; 2 Nordic Center for Earth Evolution, University of Southern Denmark, Odense, DK Denmark; 3 The Brady Urological Institute, Johns Hopkins School of Medicine, Baltimore, MD 21287 USA

**Keywords:** Multicellularity, Evolution, Phenotypic separation, Cancer, Animals, Cambrian explosion

## Abstract

Our understanding of the rises of animal and cancer multicellularity face the same conceptual hurdles: what makes the clade originate and what makes it diversify. Between the events of origination and diversification lies complex tissue organization that gave rise to novel functionality for organisms and, unfortunately, for malignant transformation in cells. Tissue specialization with distinctly separated cell fates allowed novel functionality at organism level, such as for vertebrate animals, but also involved trade-offs at the cellular level that are potentially disruptive. These trade-offs are under-appreciated and here we discuss how the wide separation of cell phenotypes may contribute to cancer evolution by (a) how factors can reverse differentiated cells into a window of phenotypic plasticity, (b) the reversal to phenotypic plasticity coupled with asexual reproduction occurs in a way that the host cannot adapt, and (c) the power of the transformation factor correlates to the power needed to reverse tissue specialization. The role of reversed cell fate separation for cancer evolution is strengthened by how some tissues and organisms maintain high cell proliferation and plasticity without developing tumours at a corresponding rate. This demonstrates a potential proliferation paradox that requires further explanation. These insights from the cancer field, which observes tissue evolution in real time and closer than any other field, allow inferences to be made on evolutionary events in animal history. If a sweet spot of phenotypic and reproductive versatility is key to transformation, factors stimulating cell fate separation may have promoted also animal diversification on Earth.

## Introduction

Animals are capable of a large range of remarkable functions, like movement and sexual reproduction. Traditionally, we explore how such functions contribute to the evolutionary success of species. However, vertebrate animals are made of tissues that consist of cells with hundreds of different functions. These functions are defined by cell differentiation during development and tissue renewal, and again redefined in the case of cancer. Cells and cell interactions fundamentally define the evolutionary success of animals and the cancers that arise in them. To explore evolution of multicellularity broadly through the lens of tissue provides a unique perspective and informs our understanding of both the evolution of animals and of cancer. We present a complementary and orthogonal view on the evolutionary success of vertebrate animals as relying on their remarkable separation of cell phenotypes into disparate tissues, and how the violation of this separation leads to cancer.

An overwhelming majority of cells in the human body are somatic with a developmentally defined fate that, under most normal circumstances, requires them to stay sessile and interact with their neighbour cells. The non-motile, non-replicating, and highly differentiated cell phenotypes are rigid, or “petrified”, and play a limited role in the evolutionary processes. In most tissues these petrified cells are replenished by a smaller pool of immature and still motile stem cells with phenotypic plasticity. The dichotomous interplay between cell phenotypic plasticity and petrification is a pillar in the framework of complex multicellular organisms, but both its evolution and devolution remain poorly understood.

Cancer represents a violation of the evolutionarily defined phenotypic separation of cells, as an increasing pool of cells regain motility and replicative capacity. This transformation of tissue represents a revolutionary speciation event within the human patient. Indeed, compared to the about two dozen rises of persistently multicellular organisms (clonal, aggregative or embryogenic) in the billion-year-old history of life on Earth, the tens of millions of cases of human cancer every year can be regarded as the most successful multicellular events on Earth.

## Cell specialization trade-offs

The specialization of animal tissues during development involves epigenetic controls on genes to successively limit a cell’s phenotypic options [1, 2]. These early epigenetic programmes distinguish the germ layers of endo-, meso-, and ectoderm, and ensure that cells along these lineages differentiate appropriately. With cell differentiation comes the organism-level evolutionary reward of specialized cell types, tissues, and organs. Conversely, the risks associated with petrified cellular phenotypes are not well defined and rarely considered. As cells progress further on the differentiation continuum, cell plasticity is traded for cell specialization. What are the trade-offs for widening the separation between cell phenotypes that are versatile versus petrified?

**Fig. 1:**
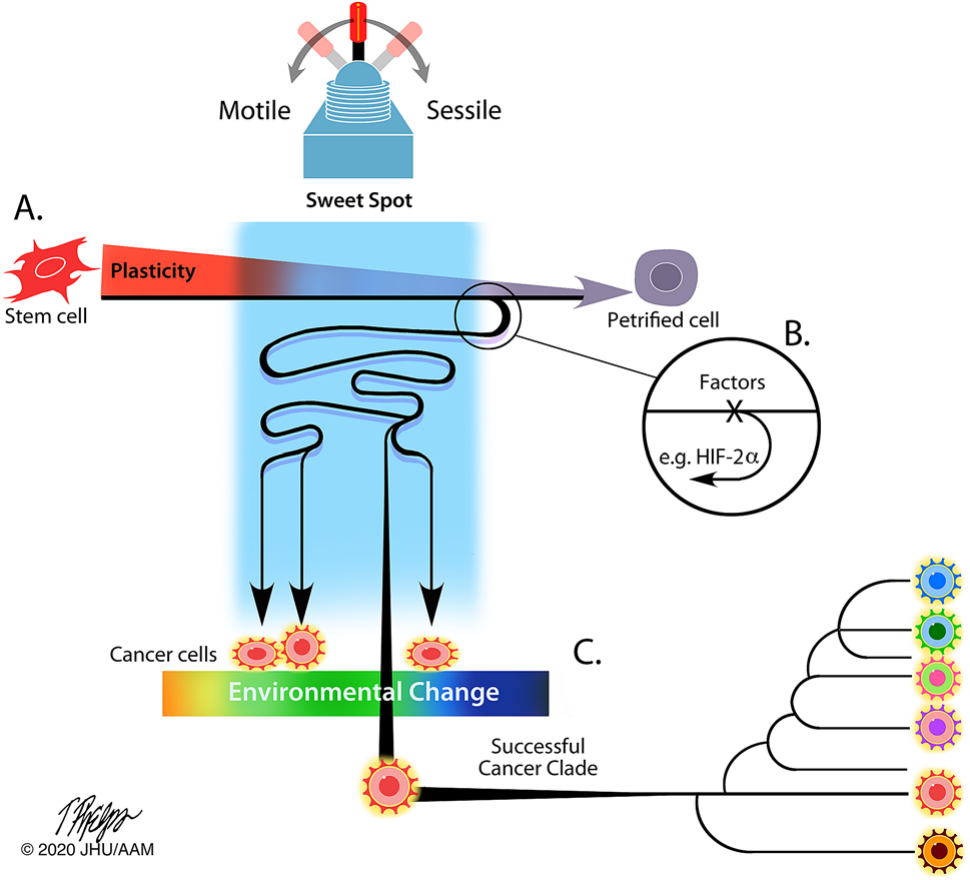
The modification or toggling of the wide separation of cell phenotypes occurs within an ‘sweet spot’ (blue field) along the continuum of cell fate definition and differentiation. **a** Cells progress on the differentiation continuum where cell plasticity and motility are traded for cell specialization and sessility. **b** Petrified cells regain access to genetically and epigenetically locked programmes via factors, like HIF-2α, during wound healing but also hijacked in cancer evolution. **c** Out of the those with higher evolvability (phenotypic plasticity within the sweet spot that are also heritable), continuously changing environmental conditions (multi-coloured barrier) selects for the most versatile phenotype that also diversify into the cancer clade

In vertebrate tissue, individual petrified cell phenotypes have lost the capacity for passing on phenotypic variation (evolvability) and are evolutionary bystanders. The diversity of differentiated cell types allows a variety of remarkable tissues that assist organisms to survive, but the non-dividing somatic cells per se do not assist animals to genetically adapt to changing environments (Fig. 1a). This is not the case in all forms of multicellular life. For example, multifunctional cells in primitive animals, some green algae (and plants), and fungi can continuously change their phenotype in a process known as trans-differentiation. Sponge cells, for example, transdifferentiate continuously and remain in the same fate for as short at 2 h [3]. The change in the phenotype of a sponge cell can occur in response to changes in environmental oxygen concentrations or nutrient availability [4]. The cells’ capacity to continuously change their phenotype assists the organism to quickly adapt to changes in environmental conditions. Notably, organisms with such trans-differentiating multifunctional cells have relatively few cell types and a limited number of tissues [5]. For example, fungi, macroalgae, and cnidarians have at most ten cell types that are often multi- or totipotent [6, 7]. Thus, with the cell phenotype never terminally differentiated and the ‘tissue’ never very specialized, rapid cell fate switching allows the organism to maintain versatile adaptability. This capacity for cellular adaptability is exaggerated at the organismal level when combined with asexual reproduction (e.g. budding). For the organism, the cellular adaptability together with asexual reproduction now allows swift and heritable responses, i.e. high evolvability. In contrast to the high evolvability of a cnidarian or sponge, the bulk of cells in vertebrate tissue are evolutionary bystanders, and therefore organismal evolvability is mediated differently. With the separation between cell phenotypes in vertebrate tissues, organismal adaptability does not invoke the versatile adaptability of rapid shifts of cell fates. The evolutionary power is instead allocated to a few specific pools of stem cells and through sexual reproduction.

Cells with phenotypic plasticity have the capacity to sustain both the species (through germ cells) and the tissue (through stem cells) [8]. Thus, in vertebrates it is clear that there is a trade-off between tissue specialization and a particular individual cell’s contribution to the evolvability of the organism as a whole. Cells with phenotypic plasticity have the capacity to evolve novel functions, allocating evolutionary power to a relatively few numbers of cells. With the role of evolvability granted to a few sourcing stem cells, corruption within this small pool of cells carries the risk that any errors will affect the whole organism of largely differentiated cells. Indeed, a hallmark of cancer cell fitness is how epigenetic shifts favour cell stemness over cell differentiation [9]. Cancer incidence has been modelled as a function of the number of stem cells and their rate of cell division [10]. ‘Stemness’, however, represents an evolutionary benefit for as long as stem cell function remains developmentally defined, and only becomes an evolutionary liability if that definition is lost. The corruption of this definition may lead to novel cellular adaptability or, if heritable, increased evolvability as observed in the cancer clade [1, 11]. Unfortunately, this gained capacity of cellular adaptability within otherwise ‘petrified’ somatic cells does not benefit the organism. The gained capacity for evolvability only benefits the population of individual cells with the capacity for proliferation (reproduction). Therefore, cell stemness as a hallmark of cancer may be best understood in the context of the role of phenotypic reversal through factors and how, paradoxically, some tissues with a high stem cell proliferation are remarkably robust.

The capacity to modify an individual adult cell’s differentiation programme would depend on two conditions: the strength of the modifying factor and the degree of cell phenotypic differentiation. Potent factors can unlock the developmentally defined epigenetic programmes of otherwise differentiated cells. This has been shown experimentally by how a cocktail of factors like Oct4, Sox2, and Myc can induce stemness in highly differentiated cells in vitro [12]. In vivo, activation of HIF-2α induces cell stemness and de-differentiation [13–15], regardless of tissue oxygenation that otherwise would promote cell differentiation [16]. Normally, cell stemness is associated with hypoxic conditions and therefore the HIF-2-driven phenotype is called ‘pseudohypoxic.’ A factor that can induce cell plasticity at any tissue setting is certainly potent and appears to be critical during development and tissue renewal, e.g. cells express HIF-2α in a spatial and temporally restricted manner [17–20]. However, the corruption of the HIF-2 driven pseudohypoxic phenotype is common in many cancers [21]. The inappropriate activation of this type of factor within an otherwise differentiated cell releases the cell from its developmentally defined epigenetic programming. The release leads to cell stemness and an evolutionary advantage if viewed through the lens of the individual cell but is a risk to the organism as a whole. Thus, lethal cancer is the product of devolution of specialized cellular phenotypes and is a risky trade-off in the evolution of differentiated cells and tissues.

To summarize, the wide separation between phenotypically differentiated cells from cells that maintain plasticity allows vertebrate tissue specialization but also involves factors with the power to modify and violate this separation. When violated, phenotypic separation ceases to be a benefit and instead represents a liability for the organism as a whole if it endows adaptability to a cell population that also can reproduce, i.e. the cancer species.

## Cancer as a violation of the separation of cellular phenotypes

The importance of the separation of cellular phenotypes—from stem cell to petrified cell—cannot be underestimated in our efforts to understand both the robustness of the human body and the events of carcinogenesis. Generally, the separation of cellular phenotypes in the organism as a whole is so robust that the development of cancer, on a cellular level, is both difficult and rare [10, 22–26]. The human body contains approximately 30 trillion cells, the majority of which are terminally differentiated, in petrified and non-replicative states [27]. Since mutations occur in conjunction with cell division, a cell that never passes through the cell cycle has virtually no chance of becoming cancerous since it will never pass on mutations to daughter cells. It is estimated that 50–70 billion cells are replaced by the adult human body per day, generated from a small pool of non-petrified stem cells. By age 70, stem cells successfully have replaced 1–2 quadrillion (10^15^) cells. This phenomenal replicative capacity of stem cells also comprises a liability and is likely a critical factor in tumourigenesis [28]. Despite this phenomenal power of stem cells, the rise of cancer multicellularity in the human body is proportionally rare. Although cancer clones successfully break developmental programmes and pass through multiple evolutionary bottlenecks, only an infinitesimally small proportion gain replicative immortality [29–33]. An even smaller number acquires enough genetic and epigenetic modifications to develop into a lethal cancer clone, suggesting that focusing primarily on stemness as the origin for novel multicellularity is insufficient [30].

The theoretical robustness of the human body and statistical rarity of tumourigenesis at the cellular level, however, provides little comfort to a patient when cancer affects 1 in 3 men and 1 in 4 women over their lifetimes, globally killing 10 million people every year [34, 35]. Advances in our ability to predict the rise of cancers in certain tissues are urgently needed. Viewing the generation of lethal cancers through a lens of tissue transformation allows us to explore tumourigenesis in terms of a violation of the separation between cells of phenotypic plasticity and those that are petrified. This undesirable event of tissue devolution leads to the revolutionary speciation event of cancer. But what in this violation leads to malignancy?

Based on the pivotal role of stem cells for replenishing somatic tissues, it is somewhat straightforward to visualize how a hematopoietic stem cell possesses the power of evolvability that, if corrupted, leads to uncontrolled cell proliferation and devolution of the normal tissue system resulting in leukaemia. But how often does cancer arise from pluripotent stem cells? Does cancer also arise in terminally differentiated petrified cells? If so, petrified cells would have to regain access to genetically and epigenetically locked programmes via specific factors (Fig. 1b). The keys to delicate and brief adjustments of developmental programmes, such as those critical during wound healing, have been selected for over millions of years of normal animal evolution. In cancer, however, genetic mutations or epigenetic modifications can hijack these powerful factors to unlock the developmentally defined phenotypic separations [10, 26, 36, 37]. How the unlocking of developmentally defined differentiation programmes is explored and explained matters for our understanding of where cancer arises along the cellular differentiation pathway. Understanding the qualities of cancer’s cell type of origin would improve our abilities to predict and restrain the evolutionary success of cancer [1, 38–41].

If cancer originates in a moderately differentiated cell, i.e. a stem cell, it may require weaker factors to unlock or skew its developmental programmes that initiate a new cancer clade within an individual. This may explain why, in general, leukaemia and lymphomas appear to be more clonal (reflecting lesser evolvability) and why paediatric tumours have fewer mutations than cancers arising as adenocarcinomas or sarcomas from adult and solid organ tissues [37]. If cancer originates in a somatic petrified cell, the stronger factors needed to unlock the developmentally placed programmes may also unleash more evolvability as these cells can now move and reproduce. With these regained capacities, the petrified cell leaves its position as a sessile evolutionary bystander to become an initiator of a new cancer species. When regained cell plasticity also involves a capacity for motility and invasion, the cell may now both successfully seed new sites within the human body and pass on its phenotype by cell division. When regained cell plasticity also involves increased capacity for motility and invasion, the cell may now have greater success both in initial metastatic seeding of a distant site as well as passing on its phenotype by cell division. Similar to the case of sponges described previously, the fast rate of cell fate change (trans-differentiation) in combination with asexual reproduction merges cellular adaptability with heritability, leading to higher evolvability. This higher evolvability is potentially advantageous when and where environmental conditions are changing—which they are doing constantly in the human body (Fig. 1c). Ultimately, the increase in evolvability leads to hyperspeciation within the cancer clade, spread within the organism (metastases), and lethality. It remains unknown to what extent a cancer originates from a fully differentiated cell. Clearly, however, there is evolutionary power in the capacity to modify and toggle between cell phenotypes and, thus, in factors that allow the reversion of phenotypic petrification (Fig. 1).

## The proliferation paradox

Paradoxically, some animal tissues with a high stem cell proliferation are remarkably robust and do not develop cancer. Indeed, some primitive organisms’ tissues consist entirely of multifunctional cells. This challenges the idea that stem cells hold an evolutionary capacity that risks being corrupted. In the case of basal metazoans, like sponges, ctenophores, or cnidarians, their few but multifunctional cell types are either stem cells or can de-differentiate back to stem cells. The evolutionary power of their cells is demonstrated by how a clonal aggregate of just a few cells (as after blending and sieving the organism through a mesh) forms a new individual. This begs the question: if all their cells are transiently stem cells, why don’t basal metazoans get cancer all the time? Alternatively, is the rapid cellular adaptability and asexual reproduction (i.e. high evolvability) of basal metazoans analogous to cancer growth?

**Fig. 2:**
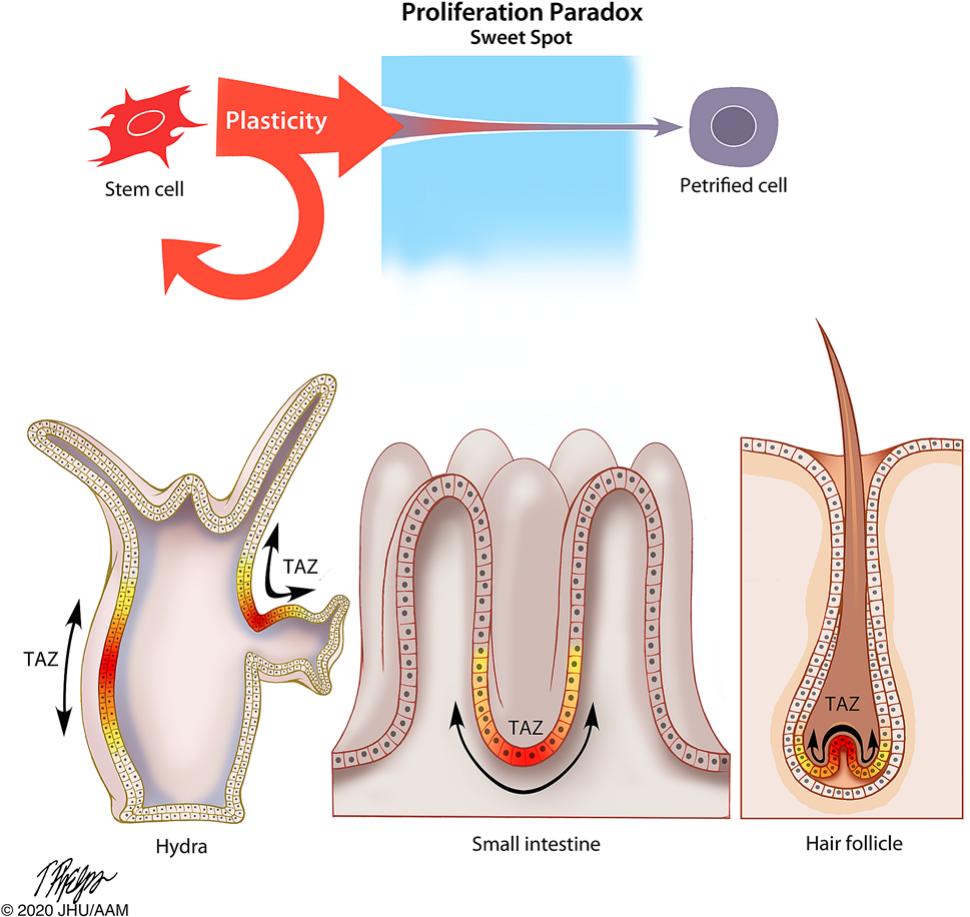
The Proliferation Paradox states that high cell turnover is uncoupled to lethal malignancy in both organisms (e.g. *Hydra*) or human tissue ecosystems (e.g. human small intestine or hair follicle). In the epithelial layers of *Hydra*, the crypts of the villi of the small intestine, and of the hair follicle, immature cells from within a transiently amplifying zone (TAZ) replenish tissue at a high rate. The limited separation of cell phenotype and the high rate of cell fate change, both, may be keys as to why these organisms rarely develop cancer

Cancer is traditionally defined as a disease in which cells anywhere in the body divide uncontrollably. Cancer initiation, therefore, is a result of how, when, and, particularly, in which kind of cell this uncontrolled cell division occurs. Cancer cells are often termed “immortal” by how they abnormally maintain proliferation [9]. However, this cellular immortality must be compared to that of tissues and organisms. For example, the cnidarian *Hydra* is described as an immortal organism because of its lack of senescence [42]. The secret of this organism’s immortality is found on the *tissue* level that constantly sources new cells near its foot and sloughs them off at the tip of its tentacles (Fig. 2). This is true tissue transformation, but a non-malignant one. The turnover of cells takes about 20 days from phenotypic plasticity to differentiation and sloughing [43, 44]. Similarly, sponge cells are immortal when aggregated (but not when unattached) and somatic cells are continuously sourced by multipotent cells [45]. It has been suggested that the separation of cell fates (into germline and soma) also determines that normal animal cells are mortal when unattached (regulated by adhesion molecules and receptors like integrin) [46]. Thus, individual cells of basal metazoans are mortal, but their collective and constant flux makes the organism immortal. The immortality rests upon dynamic cell fate turnover in their clonal tissue structure.

Basal metazoans and cancer can be considered to be analogous, in the sense of their dynamic cell fate turnover in a clonal structure. Clonal expansion and evolvability are key features to their success within an existing ecosystem. In both cases, their overall evolvability increases by how individual cells can dynamically change cell fates and thus adapt to constantly varying conditions. In cancer, this is taken one step further with individual cells regaining the capacity for reproduction and thereby can pass on cells with the versatile phenotypes. This capacity for single cell immortality was common amongst ancestral eukaryotic organisms, like protists, but is comparably scarce within tissue-grade organisms. Additionally, aggregations of the immortal replicative cancer cells demonstrate the same dynamic cell fate turnover that provide basal metazoans with high adaptability. This would suggest that cancer demonstrates immortality on both the cellular and tissue level. With one foot in both camps, the immortality of cancer cells when both aggregated, through dynamic fate turnover, and when single lead to uncontrolled growth, and patient death. Whilst the expansion of basal metazoans is controlled within its ecosystem (such as through access to food or pressure by predators), the clonal expansion of cancer is uncontrolled until its ecosystem (host) collapses.

Paradoxically, high cell turnover—proliferation—is a hallmark of some non-transformed human tissues that rarely develop cancer. Whilst epithelial cells of the small intestine have the fastest turnover rate in the human body and are replaced every 3–5 days [47], cancer in the small intestine is extremely rare (< 1% of all cancers, incidence rate of ~ 2 cases per 100,000 people per year) [35]. Similarly, human hair follicles collectively replace a vast number of cells, but hair follicle cancer is very rare (< 1% of only *benign* skin tumours) [35, 48, 49]. As noted above, the hematopoietic system replaces ~ 50 billion cells per day but the incidence of leukaemia is relatively rare (~ 3% of all cancers, incidence rate of ~ 14 cases per 100,000 people per year). Conversely, cells in the colon epithelium have low cell turnover (5–21 days) but colon cancer is the third most common cancer with an incidence of ~ 40 cases per 100,000 people per year [35]. Moreover, the tissues with the highest rates of cell turnover also have low rates of lethal cancer. Basal cell carcinoma (BCC), for example, is the most frequently occurring form of all cancers. More than 4 million cases are diagnosed each year in the USA alone. BCC most often occurs when DNA damage from exposure to ultraviolet (UV) radiation triggers changes in basal cells in the outermost layer of skin, resulting in uncontrolled cell division. The UV radiation acts as a weak transformation factor, allowing uncontrolled proliferation with little chance for further evolution into a lethal phenotype. The discordance between proliferation and stemness characteristics and the risk for uncontrolled lethal tissue transformation presents a “Proliferation Paradox”.

Cell proliferation and stemness are hallmarks of continuous tissue formation in basal metazoans, cancer, and normal vertebrate tissues (Fig. 2). In the case of basal metazoans where high proliferation sustains the entire organism, cells do not differentiate very many steps from their stem cell phenotype, but the fate of individual cells is continuously in flux. This high rate of cell fate change may be one key as to why these organisms rarely develop cancer. If this rate of cell fate change is fast, it may be too rapid for novel genotypes to develop. This suggests there may be a “sweet spot” between a proliferative versus petrified phenotype where the cellular corruption that leads to cancer occurs (Fig. 2). If diversification is a result of a change in evolvability, a result of genetic and reproductive heterogeneity, it may require both time and ‘toggling’ of both genotypes and phenotypes. The Proliferation Paradox also suggests that if cellular corruption originates in the most basal stem cells of organisms and tissue with fast cell turnover (high proliferation), there would be no exceptions to the association between high proliferation, stemness, and cancer risk; which there are. Thus, cancer may originate in cells in a sweet spot sometime after they leave ‘true’ stemness but whilst still differentiating to a ‘petrified’ phenotype.

**Fig. 3:**
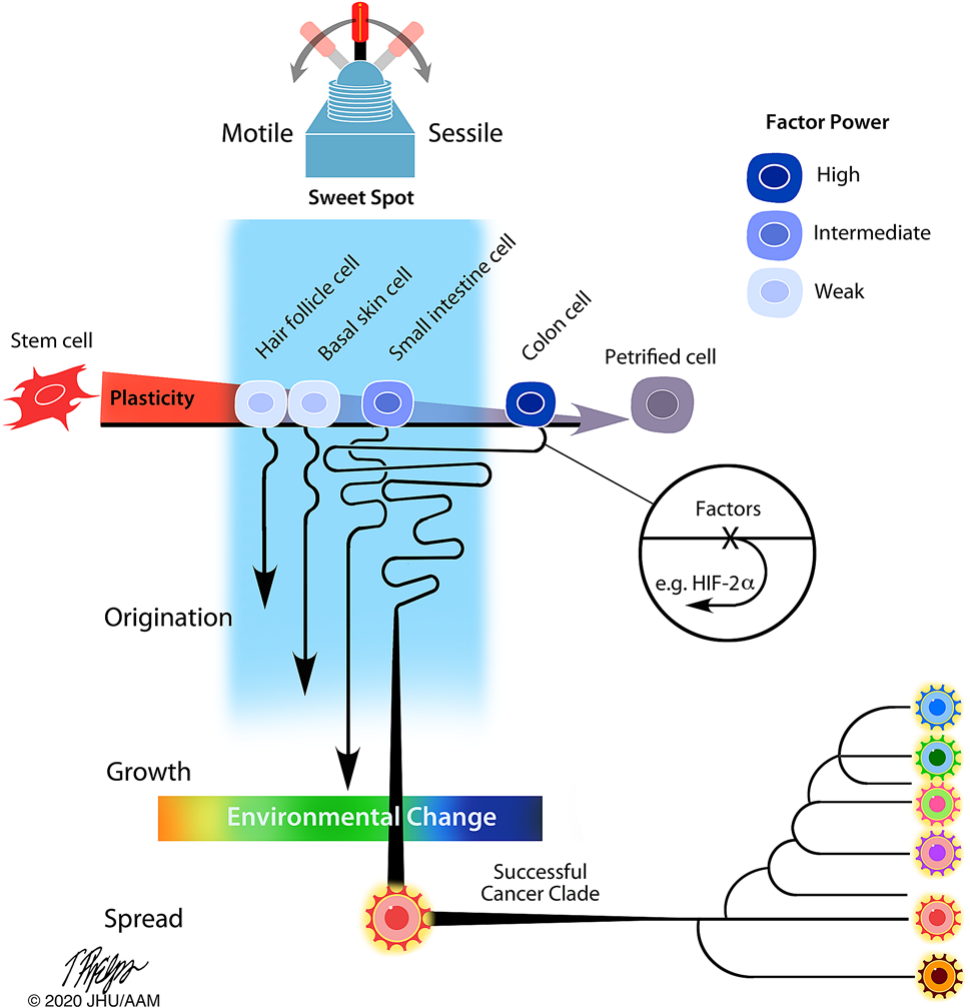
The unlocking of developmentally defined epigenetic programmes of differentiated cells requires factors with different powers. At a phenotypic separation that is narrow, weak factors unlock a reversal and, when involved in corruption, leads to a weak error (a non-lethal cancer, e.g. BCC). The reversal of a petrified phenotype requires a factor of high power, e.g. HIF-2α, where corruption leads to severe error (powerful phenotypic toggling). The corruption of factors with high power result in higher evolvability that stretch over origination, growth and to spread of the cancer clade

The ‘immortality’ of organisms and tissue with high cell turnover may lie in how their cells never move very far on the differentiation continuum. For example, the < 10 multifunctional cell types in sponges or cnidarians never differentiate far from stem cells. If the phenotypic separation of stem-to-petrified remains small, the ability of the factors at play to modify the developmental programme may be limited. For instance, the corruption of the phenotypic separation by a less potent factor may also lead to a less severe error (a non-lethal cancer, e.g. BCC). On the other hand, the wide range of cell fate separation makes the petrified vertebrate cell very constrained. The unlocking of these developmental constraints would require particularly potent factors, such as HIF-2α (Fig. 3). Notably, HIF-2α is vertebrate-specific—and so it seems are metastatic cancers [50]. Admittedly, the rate of invertebrate cancer incidence is unknown, but still it appears that their metastatic tumours would have been observed more commonly. For example, Darwin’s favourite animal, earthworms, make up the largest terrestrial animal biomass and if their incidence of metastatic cancers were as common as in vertebrates—we would have noticed.

How human tissue with fast cell-fate turnover like the small intestine avoids the frequent development of cancer remains cryptic. The development of the small intestine involves dramatic remodelling and de-differentiation of cell phenotypes, as more non-proliferative (petrified) cells become proliferative [51]. Thus, the versatile cell differentiation hierarchy of the developing small intestine suggests that factors unlock developmental and epigenetic controls, evoking evolutionary programmes. The capacity for evolvability by modifying phenotypic separation may be different in the adult large intestine. The base columnar stem cells are known to continuously feed new cells to the lining, but from there, cells appear to get a one-way ticket to differentiation, phenotypic petrification (e.g. goblet cells) and being sloughed off [52]. The cell differentiation hierarchy of the post-natal small intestine resembles that of the rapidly proliferating cell trans-differentiation hierarchy of the basal metazoans. If evolvability encompasses a sweet spot of cellular adaptability *and* the onset of proliferation through the loss of differentiation epigenetic programming, the onset of asexual reproduction may be inaccessible to cells in the small intestine epithelium since cells are sloughed off within a few generations of differentiation.

In summary, high cell turnover associates with tissue transformation, but not always with lethal malignancy in either organisms (e.g. *Hydra*) or in human tissue ecosystems (e.g. human small intestine). This could suggest that there is a sweet spot along the phenotypic continuum where genotypic origination manifests itself, for potentially subsequent diversification. This particular manifestation would occur beyond a true stemness (or else we would see a high rate of cancer in the hair follicle and small intestine) and before (or after the reversal of) phenotypic cell petrification. Therefore, high turnover of cells and their fate may protect against uncontrolled clonal diversification by either speed (no time) or by the inaccessibility of potent transformation factors to modify the most petrified cells.

## Clues from tissue structure advance our models of animal evolution

Revolutionary organismal events on Earth are rare in comparison to that of cancer. Only twice have complex multicellularity with organ systems evolved to persist and diversify: animals and plants. Current models of these two organismal revolutionary events deliberate on environmental, ecological, and genetic parameters [53–56]. To a much lesser extent are models shaped by characteristics and selection of cellular phenotypes as observed within tissue and during its transformation, i.e. cancer. This is surprising since multicellular organisms are composed of evolving tissues. We suggest that specific constraints around tissue formation, renewal, and transformation in modern animals may reflect clues to the evolution of multicellularity broadly. To extract such clues, a framework of phenotypic separation is particularly useful.

The framework of phenotypic separation suggests (a) that the wide phenotypic separation is central but risky to the function of complex multicellularity and (b) that the capacity for phenotypic toggling contributes to increased clonal evolvability, cladogenesis, and clade diversification (hyperspeciation). For the animal kingdom, cladogenesis and hyperspeciation is apparent from the transition to the Cambrian Period some 543 million years ago and was a dramatic evolutionary event [57]. (Green plants also started to diversify in this time interval, although trees and other vascular plants on land diversified later [54]). In common with hyperspeciation within the cancer clade, the diversification of animals was fast (geologically speaking) and is linked to increased evolvability and motility. Within a few tens of millions of years, all animal phyla evolved and, since then, animal body plans have remained largely fixed. Similar to the hyperspeciation of cancer clade, drivers behind the Cambrian explosion of animal diversity remain cryptic. Neither geological nor biological evidence can fully support the idea that the event was triggered by one factor, such as increasing atmospheric oxygen or genetic novelty [7, 55, 58, 59].

**Fig. 4:**
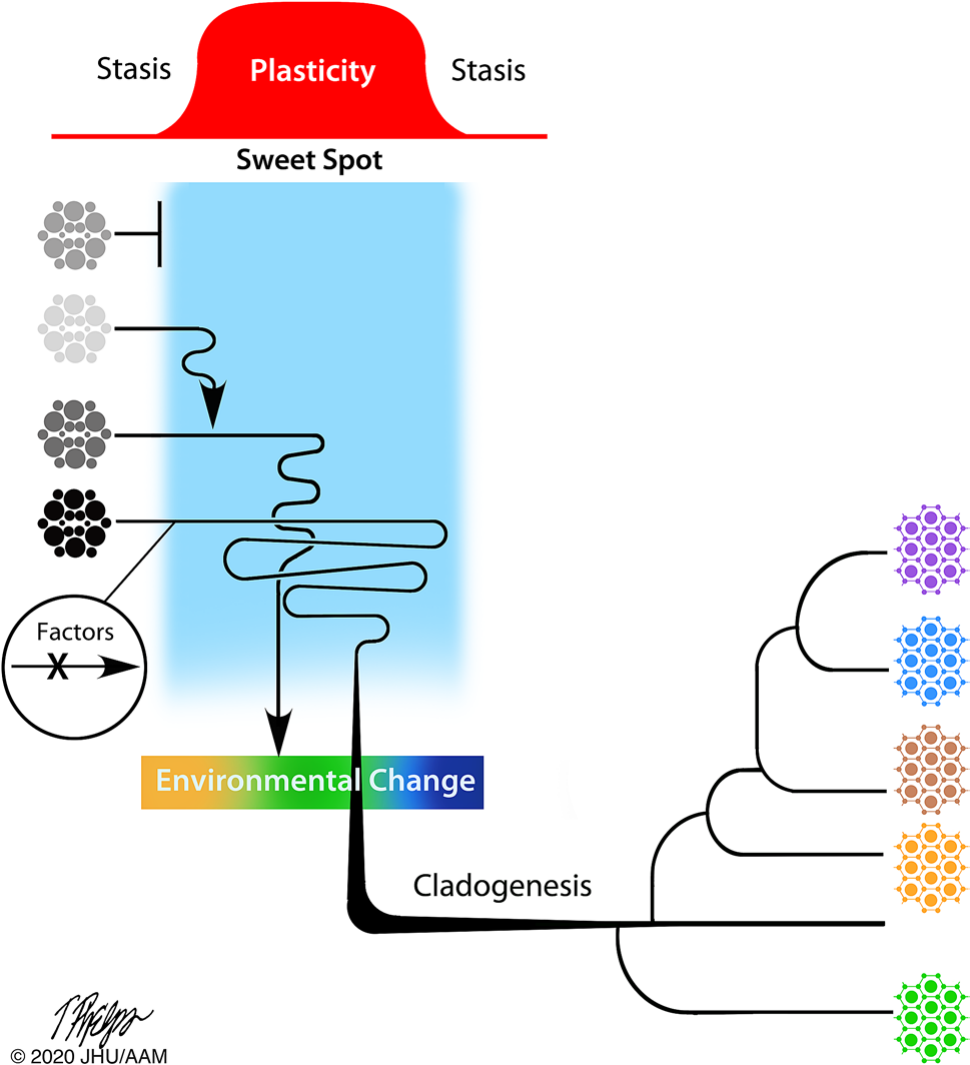
Phenotypic toggling within a sweet spot between two phases of morphological stasis is (by analogy to tumourigenesis) inferred equally important for the transformation of complex multicellular organisms (e.g. animals or plants). Clonal organisms with high cell rate turnover (grey, left of sweet spot) remain in morphological stasis until factors permits a wider range of phenotypic cell fates and phenotypic toggling. With unleashed organismal evolvability (sweet spot; blue field), constantly changing environmental conditions (multicoloured barrier) select for those with the refined capacity to sense and respond accordingly. These organisms subsequently diversify (species symbolized by tissues in different colours, to the right). The window of high evolvability—tissue transformation—is transient and after diversification, tissue morphologies (body plans) remain largely the same (stasis)

Tumourigenesis as an intra-organismal event is analogous, in some respects, to the revolutionary organismal event when animals diversified on Earth. Analogous observations within modern animal and tumour tissue allow us to infer that the capacity for phenotypic toggling and heterogeneity *itself* lead to increased options such as motility and reproduction, which thus leads to higher heritable adaptability (evolvability). This framework differs from models in which initial environmental change selects for phenotype variability. For example, the importance of the flexible animal phenotype for evolution has been demonstrated but generally as selection during environmental change [60]. Our view that an onset of multicellular evolvability first relies on phenotypic and reproductive toggling (rather than first requiring genotypic innovation or initial environmental change) has two implications for models of the origination and diversification of animals on Earth. First, early metazoans (proto-animals) may have been resilient to transformation. Second, the Cambrian explosion may have been the result of proto-animals transiently accessing an evolutionary optimum, or sweet spot, of phenotypic toggling that, in combination with ecosystem heterogeneity and increased heritable adaptability (evolvability), changed the animal kingdom once (Fig. 4).

The Proliferation Paradox suggests that tissue turnover can be too fast for any novel species to originate. Basal metazoans like *Hydra* (analogous to tissue homeostasis and resilience as the human small intestine or hair follicles) could be an evolutionary ‘one-way street’ that lack access to the sweet spot for the success of genotypic experimentation (transformation). Did organisms with similarly robust but non-evolvable tissue structure exist before the Cambrian Explosion? Soft organisms like cnidarians and ctenophores are poor candidates for fossilization, but within the Ediacaran biota of ‘proto-animals’ some fossils are worth noting. The Ediacaran biota of multicellular marine organisms, some of them likely the ancestors of animals, are known to have been present for up to ~ 90 million years before the Cambrian explosion [61, 62]. In this biota, fossil evidence interpreted as both primitive ctenophores [63] and sponges [62] are described, but also the sessile Rangemorphs organisms of a frond-like cryptic symmetry that remained robust for some 30 million years [64]. Also, molecular clock estimates suggest that sponges (clonal organisms) existed ~ 100 million years before their fossils are found in the Cambrian [65, 66]. A gap of ~ 100 million years between the rise of basal metazoans and the diversification of Eumetazoa is a debated conundrum [67, 68]. The role of tissue architecture is increasingly discussed as a driver behind the transition from eukaryotic unicellular to multicellular diversity, when the regulation of cell fates is presumed to have changed from being temporal (different life stage) to spatiotemporal (within tissue) [69]. Tissue architecture can, however, be robust and the Proliferation Paradox is a potential explanation for such the gap between the rise of basal metazoans and the diversification of Eumetazoa. The resilience of fast tissue turnover may not have offered a particularly potent ‘fuse’ for further animal diversification. Therefore, an evolutionary stasis in the presence of basal metazoans is not unexpected.

The power of phenotypic toggling further suggests that the separation into cell plasticity or petrification leads to robust tissue specialization, which can be broken by factors that have the potential and timing to create heterogeneity and instability. In modern vertebrates, one such factor could be HIF-2α, associated with cancer-related corruption of cell stemness and unique to vertebrates [58, 70]. In the Cambrian and for invertebrate animals with less petrified cells, however, less potent factors could also have had the power to modify the phenotypic and ecological landscape. HIF-1α, for example, could be one such candidate. HIF-1α is a short-lived factor, associated with fundamental phenotypic changes and expressed in all modern eumetazoans except sponges and ctenophores [58, 70–72]. A tissue factor that resulted in an increased capacity for phenotypic toggling and heritable adaptability (evolvability) could have had wide-reaching effects for simple multicellular organisms. This could allow the step from a clonal organism with controlled and fast tissue renewal to organisms with more specialized tissues. The ecological effects of phenotypic toggling and increased evolvability, however, are transient and potentially advantageous only when environmental conditions are in flux. Tissue with perfectly robust petrified cells will not be able to undergo tissue transformation. Without novel tissue transformation, the organismal ecosystem would also stay static. Indeed, that most of a species’ history is in stasis with stable morphology and how dramatic physical events can punctuate this equilibrium [73, 74] is much discussed. Here, we simply suggest that a dramatic physical event is that of chemical conditions in flux—which is common both on Earth’s surface and in the human body. Both the initial hyperspeciation of animals and plants and hyperspeciation events after numerous mass extinctions can be argued to associate with circumstances where either environmental or biological conditions are in flux, i.e. varying ocean chemistry [75] or predation pressure [76]. The recovery after mass extinction may resemble a relapse in cancer rather than a *revolutionary* organismal event, and both may demonstrate how populations with traits of phenotypic toggling and evolvability hold the evolutionary advantage under dynamic settings. In contrast, long periods over animal and plant history can be regarded as static. Thus, phenotypic separation and increasingly sessile cells allowed tissue specialization that, on one hand, allowed controlled organismal motility though muscles and advanced nervous systems but, on the other, reduced cellular motility and clonal evolvability, thus creating evolutionary bystanders.

Clues from tissue organization, therefore, may advance our understanding of animal diversification by highlighting the role, robustness, and vulnerability of phenotypic separation. Characteristics of phenotypic separation in vertebrate organ and tumour tissue suggest that along the continuum towards petrification and after leaving a basal stem cell state, tissue and cell fate turnover can be too fast to let genotypic innovation manifest itself. This would suggest that clonal organisms with fast tissue and cell fate turnover like sponges or cnidarians existed for eons without diversification; an observation at least not contradicted by the geological evidence. In a phase along the continuum of phenotypic petrification, factors that increase heterogeneity (motility, reproduction) and heritable adaptive forces (evolvability) could lead to transformation of both ecosystems (organisms) and tissue (cancer). This phase, however, may have a sweet spot since the specialization of tissue, in itself, reduces evolvability again. The ultimate implication of the petrified cell phenotype and tissue specialization such as observed in vertebrates is that evolvability is deemed to be reduced. Only within the evolvability sweet spot *and* when environment chemical conditions are in flux will the species or kingdom diversify.

## Conclusions

The fields of cancer biology and geobiology have the same two conceptual hurdles: what makes new clades originate and what makes them diversify. A framework of phenotypic separation—where the most extreme cell phenotype is petrified, sessile, and an evolutionary bystander—provide both fields with novel and relevant clues:

1. The capacity to toggle cellular phenotypes may lead to a broader repertoire of cellular motility and reproduction. If this clonal heterogeneity also leads to heritable adaptability (evolvability), novel genotypes may manifest and diversify.
2. The Proliferation Paradox demonstrates that some tissue and organisms maintain control of high cell proliferation and stemness. These tissues, however, have a high rate of cell fate turnover and the shedding or sloughing of cells that may lower the probability of tissue transformation that would be evidenced by either organismal evolution or the organism developing cancer. The paradox suggests that there is an optimum between the true stem cell phenotype and a terminally differentiated petrified cell where higher evolvability can occur. An implication of this paradox is demonstrated by the fact that clonal organisms like sponges have persisted without diversification. In the cancer field, it implies that the association between lethal proliferation and adult cell stemness may need revision.
3. The evolutionary pivot point in the capacity of phenotypic toggling suggests that factors with the potential to revert phenotypic petrification can alter the power balance within tissues. In cancer, one such factor, such as HIF-2α, could violate phenotypic separation of our tissues, giving the evolutionary power (through motility and asexual reproduction) to allow the development of a new tumour multicellularity. For animal diversification, factors, more short-term and ‘weaker’ than HIF-2α, such as HIF-1α, could have given organisms an evolutionary advantage through the ability to control phenotypic separation by bringing cells towards stemness or petrification.
4. The petrified cell phenotype is a trade-off. As in a Faustian bargain where a contract with the devil trades the free soul for the access to worldly pleasures, tissue specialization traded cellular, clonal, and organismal evolvability for organismal complexity and life in a broad range of global niches. The specialization came with the risk of cancer. In animal history, the trade-off means that the Cambrian explosion was not only a late and rare event, it would not likely occur again. In cancer, this means that induction of petrification could take back evolutionary power from the cancer clone.
5. The dichotomy between organisms with robust tissue turnover, like *Hydra*, and those when species morphology is petrified (in stasis) emphasize that also the diversification of organisms may also occur within a sweet spot of the evolutionary continuum. Species or clades will only diversify when a population accesses heritable phenotypic toggling *and* when environmental conditions in flux impose a selective pressure.

Evolutionary theory rarely focuses on what is lost as complexity increases. With the complex tissues of vertebrates where most cells are evolutionary bystanders, the capacity for phenotypic versatility should be lost or extremely restricted after development. Cancer cells, however, may access the exact same capacity when factors retract cells from a destined petrified fate. Since tissues with high cell proliferation can be paradoxically robust, we argue that the reversal of the differentiated petrified cell phenotype provides the higher risk. Also lost with complex tissues where sessile cells are opted to interact with their neighbours is the gain with single cell reproduction. The cell population with the least complex networks or dependency to its neighbours will benefit the most if new traits are heritable through asexual reproduction. Critical for the transformation of tissue therefore would be (a) the balance of cells with distinctly separated phenotypic plasticity or petrification, (b) the power of factors that can reverse the petrified cellular phenotype, and (c) that population with least dependency on neighbours will gain from increased reproductive capacity. These three components could play a role in the transformation of both tissue and species.

We anticipate that transdisciplinary explorations to understand the networks that lead to a wide separation of phenotypes of cells or species will allow us to view the evolutionary roots and cost–benefit of complex multicellularity through a new lens. This view may lead to novel grouping or divisions of tissue and organisms. For example, clonal eukaryotic organisms with high cell fate turnover could be divided into controlled (e.g. sponge or hydra) and uncontrolled (cancer) tissue. This view may also force us to study mechanisms of uncontrolled tissue transformation (cancer) as *necessary* for the transformation of the animal or plant kingdoms. For example, what if animal evolution was pushed from one stasis (where a low diversity of organisms had robust morphologies e.g. sponges or hydra) to another (where a high diversity of animals had robust morphologies e.g. animals) in part via organisms with the capacity for heritable phenotypic toggling that allowed them to bypass the ecological pressure from neighbouring organisms? A focus on the nature and modification of the petrified cell phenotype also highlights that we still do not know in what cells, or with what factors, this shift (with subsequent carcinogenesis), is induced. Ultimately, developing a tissue-perspective may inform what leads to revolutionary organismal events, whether in us or on Earth.
